# Association of Chronic Pain Severity and NSAID Use with Self-Reported Kidney Risk Indicators: A 1-Year Cohort Study

**Published:** 2026-05-20

**Authors:** ALI RAZEGHIANOUGHAZ, ANEEZA HAMEED, SAUD GUL, GHINA AL AKOUM, NIAM ADAM ABUBAKR MAHDI, SADAF SHAIKH, AYESHA SAEED, MEHREEN NAWAZ KHAN, SALMAN MUSTAFA MOHAMMAD ABABNEH, OMAR A. BARAKAT, ZIAD ABDALLAH HUSSEIN MALKAWI, ZAID ABDALLAH HUSSEIN MALKAWI

**Affiliations:** 1 Department of Internal Medicine, Medway NHS Foundation Trust, Gillingham, Kent, United Kingdom; 1 Department of Internal Medicine, Medway NHS Foundation Trus, Gillingham, Kent, United Kingdom; 2 Department of Internal Medicine, Wah Medical College, Wah Cantt, Pakistan; 2 Department of Internal Medicine, Wah Medical College, Wah Cantt, Pakistan; 3 Department of Medicine, Royal Derby Hospital, Derby, United Kingdom; 3 Department of Medicine, Royal Derby Hospital, Derby, United Kingdom; 4 Department of Acute Medicine, Hampshire Hospitals NHS Foundation Trust, Hampshire, United Kingdom; 4 Department of Acute Medicine, Hampshire Hospitals NHS Foundation Trust, Hampshire, United Kingdom; 5 Department of Internal Medicine, Hull University Teaching Hospitals NHS Trust, Hull, United Kingdom; 5 Department of Internal Medicine, Hull University Teaching Hospitals NHS Trust, Hull, United Kingdom; 6 Department of Medicine, George Eliot Hospital, Nuneaton, United Kingdom; 6 Department of Medicine, George Eliot Hospital, Nuneaton, United Kingdom; 7 Department of Internal Medicine, Fatima Jinnah Medical University, Lahore, Pakistan; 7 Department of Internal Medicine, Fatima Jinnah Medical University, Lahore, Pakistan; 8 Department of Lifestyle & Integrative Medicine, The University of Arizona, Tucson, Arizona, USA; 8 Department of Lifestyle & Integrative Medicine, The University of Arizona, Tucson, Arizona, USA; 9 Department of Medicine, Al-Yarmouk University, Irbid, Jordan; 9 Department of Medicine, Al-Yarmouk University, Irbid, Jordan; 10 Department of Medicine, Near East University, Nicosia, Cyprus; 10 Department of Medicine, Near East University, Nicosia, Cyprus

**Keywords:** chronic pain, NSAIDs, self-reported kidney risk, medication adherence, kidney risk

## Abstract

**Objectives:**

Chronic pain usually results in prolonged use of NSAIDs, thus raising the chance of experiencing self-reported kidney risk indicators. The present study examined the relationships between pain intensity, NSAID-related compliance patterns, and patients' self-reported kidney risk indicators over 12 months.

**Methods:**

A one-year prospective cohort study was conducted involving 300 adults with chronic pain. The parameters of pain severity (Brief Pain Inventory), NSAID adherence behaviour (Medication Adherence Rating Scale), and self-reported kidney risk indicators were evaluated at the start of the study and at 3, 6 and 12 months. Mann-Whitney U, Kruskal-Wallis tests, Spearman correlations, and repeated-measures analysis were utilised.

**Results:**

During the whole period, the patients who reported the highest pain severity had a strong correlation with a greater risk of NSAID adherence and self-reported kidney risk indicators (p < 0.001). Women exhibited much higher pain severity, adherence risk, and kidney risk compared to men (p < 0.05). The oldest age group (≥ 50 years) received the highest scores for all outcomes. Long-term analysis revealed small but significant increments in pain, adherence risk, and kidney risk over the year (p < 0.001).

**Conclusions:**

The association between chronic pain intensity, poor adherence to non-steroidal anti-inflammatory drugs (NSAIDs) and an increase in self-reported kidney risk indicators was observed over time. It is suggested that older people and women might need to receive more frequent checks and also be given the proper instructional material to prevent renal side effects during long-term pain management.

## Introduction

Chronic pain, which persists beyond six months, is a complex neurophysiological disorder that has a profound impact on the daily functioning and quality of life of the patients, which in most instances, leads to disability, depression, fatigue and sleep disturbances^1, 2)^. Worst pain is always linked to more functional disability and worse overall health outcomes^3)^.

Chronic pain is equally common among chronic kidney disease (CKD) patients, and it is reported that about 60% of hemodialysis patients experience chronic pain, and more than 40% patients report moderate to severe pain^4)^. NSAIDs, such as cyclooxygenase-2 (COX-2) selective agents, can be used to treat chronic pain and inflammation. Still, they have varied gastrointestinal (GI), renal, and cardiovascular (CV) safety profiles in older adults and those with comorbidities^5, 6)^.

Acute kidney injury (AKI) is a clinically significant condition due to its high morbidity and high healthcare expenditures, particularly in perioperative and medically complex groups^7)^. NSAIDs are a well-known risk factor of AKI, and the risk is increased in individuals with low initial glomerular filtration rate (GFR) or hypoalbuminemia. There is some evidence that non-selective NSAIDs are more dangerous than COX-2 selective inhibitors, including celecoxib and rofecoxib^8, 9)^.

Knowledge of these associations is essential for early detection of individuals at risk and for formulating safer pain management measures. The proposed study is intended to explore the connection between the severity of chronic pain, NSAID use, and self- reported kidney risk indicators at 1-year follow-up, which will inform both clinical practice and patient education on the safe use of NSAIDs.

### Study gap

Chronic pain usually causes people to use NSAIDs to relieve symptoms, but with time or frequent use of the drugs, they may harm kidney function. Adults may self-manage chronic pain with limited medical oversight, making early self-reported signs of kidney stress more likely to be overlooked. Meanwhile, the intensity of chronic pain can also define the prevalence and dosage of NSAID-based treatment, which may increase susceptibility to the early warning signs of self-reported kidney risk indicators. Nevertheless, there is little literature on the interaction between pain severity and NSAIDs to measure the self-reported factors of kidney risk in the real-life context. This research fills a gap by examining these associations over 1 year to understand better factors that may predispose people to kidney injury during chronic pain management.

### Objectives

The purpose of the study is to investigate the interaction between the severity of chronic pain and the use of NSAIDs, and modify self-reported risk factors of acute kidney injury, with a 1-year follow-up. In particular, it aims to investigate whether the severity of chronic pain is related to increased use of NSAIDs and whether this use is linked to early renal warning symptoms reported by the participants. The study will also examine whether chronic pain severity alone predicts these self-reported kidney risk factors, offering better insight into the factors that may contribute to susceptibility to acute kidney injury among adults with chronic pain.

## Methodology

### Study design and setting

This was a 1-year prospective cohort study carried out in adults who had chronic pain. Participants were recruited in community and outpatient settings, and follow-up assessments were done at baseline, 3 months, 6 months, and 12 months.

### Participants

Individuals aged 18 years and above who reported having chronic pain that persisted for six months or longer were eligible to be included. Patients with a known kidney disease diagnosis, hospitalised within three months due to renal problems, or having contraindications to NSAID use were excluded.

### Sample size

The population was assumed to be large in this study since there is no evidence on the number of adults with chronic pain in the general population who report self-indications of kidney risk. The estimated sample size was sufficient to have adequate statistical power to identify relationships between the severity of chronic pain and NSAID use and self- reported indicators of kidney risk. It was assumed that the confidence level and the statistical power were 95% with a possible 10-15% attrition rate during the 1-year follow-up. In this regard, and given feasibility, the study recruited 300 participants. Participants were enrolled consecutively in both community and outpatient settings until the target number was reached.

### Data collection and measures

Structured questionnaires and standardised scales were used to collect data on participants' detailed characterisation, pain severity, medication adherence, and self-reported risk of kidney disease.

### Demographic form

The demographic data were collected using a structured questionnaire that included information on age, gender, occupation, comorbidities, duration of chronic pain, and other related features. This form was intended to create a precise study design that would obtain baseline information on the participants and the contextual factors that might contribute to the use of NSAIDs and, consequently, the risk of kidney damage.

### Brief pain inventory-short form (BPI-SF)

The BPI-SF was created in 1994 by Cleelen and associates and included assessments of pain intensity and its influence on daily life. There are nine items on the scale: 4 measuring the intensity of pain and 5 measuring the disruption of pain to normal activities. Its validity, reliability, and widespread use in research for assessing chronic pain and related functional restrictions made the scale a good choice^10)^. The use of this scale allowed the evaluation of the pain level to be tested in its relationship to NSAID usage and kidney risk factors.

### Medication adherence rating scale (MARS)

The Medication Adherence Rating Scale (MARS) is a 10-item self-report questionnaire developed by Thompson et al. (2000) to assess medication adherence behaviours, including both intentional and unintentional non-adherence. In the present study, the scale was adapted specifically to evaluate participants’ adherence to NSAID therapy, including dosage, frequency, and timing of intake. This adaptation allowed the study to investigate the association between chronic pain severity and NSAID adherence patterns. It is important to note that, as a self-reported measure, responses may be influenced by recall bias or social desirability^11)^. The scale was selected for its ease of use, prior evidence of validity, and ability to provide insights into how medication-taking behaviour might relate to self- reported kidney risk.

### Self-reported kidney risk checklist

A self-reported kidney risk checklist for the study was created to assess early, self-reported indicators of kidney risk in patients taking NSAIDs. The checklist includes questions about decreased urine output, swelling of the hands and feet, unusual tiredness, and other typical signs of kidney stress. Its primary purpose was to select participants for this research, given that there is no validated self- report tool for monitoring self-reported kidney risk at the community level. This tool is non-validated and was intended only to assess participants’ perceived kidney risk, not to diagnose actual kidney injury. No formal reliability testing or clinical verification was conducted. The checklist allowed the study to explore associations between chronic pain, NSAID use, and self-reported kidney risk over the 12-month follow-up.

### Procedure

At baseline, the scales were administered to participants, who were then followed up at 3-, 6-, and 12-month time points. To obtain prompt replies, reminders were dispatched beforehand for the follow-ups. Data completeness was monitored, and changes in NSAID use and self-reported kidney risk were detected over the study duration.

### Ethical considerations and data analysis

The research was carried out in line with ethical principles for human research. The Institutional Review Board of Royal Derby Hospital (RDH-R&D/REC/2024-IM-0198) granted ethical approval. Written informed consent was obtained from all participants, and their personal and health data were kept confidential throughout the study. For analysing the data, we first applied descriptive statistics to give an overview of the demographic features of the participants, amounts of chronic pain, risk of medication adherence and self-reported kidney risk. Mann-Whitney U tests were used to assess group differences by gender. To test the relationships among pain severity, medication adherence risk, and self-reported kidney risk, correlational analyses were conducted. Multiple regression models were used to identify the strongest predictors of kidney risk. Also, a Repeated-Measures ANOVA was employed to evaluate within-subject changes in pain severity, medication adherence, and self-reported kidney risk across the four time points (baseline, 3, 6, and 12 months). Assumptions of sphericity and approximate normality were checked before conducting ANOVA. Nonparametric tests (Mann-Whitney U and Kruskal-Wallis) were used for between-group comparisons because the distributions of demographic and clinical variables were nonnormal.

## Results

### Demographic and clinical characteristics of the study participants

Table 1 indicates that the sample was predominantly older adults, with 116 participants (38.7%) aged 60 years and above, and 95 participants (31.7%) aged 50-59 years. The proportion of females was higher than that of males, with 182 participants (60.7%) versus 118 participants (39.3%). In terms of education, primary education and secondary education had the highest proportions of participants at 86 (28.7%) and 86 (28.7%), respectively. Homemakers had the most significant number of participants, at 73 (24.3%), followed by employed people at 72 (24.0%) and students at 69 (23.0%). Regarding BMI, 86 (28.7%) respondents were overweight, and 80 (26.7%) were obese. Smoking status revealed that 133 (44.3%) were current smokers. Over half of the sample had at least one comorbidity, and 175 participants (58.3%) reported any medical issue, most commonly diabetes (90 participants, 30.0%) and heart or kidney disease (74 participants, 24.7%) each. The duration of chronic pain was reasonably balanced, with a majority of 74 participants (24.7%) having less than 6 months of chronic pain. The majority of the respondents with chronic pain were headache/migraine, with 106 participants (35.3%), then joint pain/arthritis, with 93 respondents (31.0%).

**Table 1:** Demographic characteristics of participants (N = 300)

Variable	f	%		Variable	f	%
Age				Overweight (25-29.9)	86	28.7
18-29 years	20	6.7		Obese (≥ 30)	80	26.7
30-39 years	22	7.3		Smoking status		
40-49 years	47	15.7		Never smoked	63	21.0
50-59 years	95	31.7		Former smoker	104	34.7
60 years or above	116	38.7		Current smoker	133	44.3
Gender				Comorbidities	175	58.3
Male	118	39.3		Hypertension	27	9.0
Female	182	60.7		Diabetes	90	30.0
Educational Level				Heart disease	74	24.7
No formal education	36	12.0		Kidney disease	74	24.7
Primary (1-5 years)	86	28.7		None	35	11.7
Secondary (6-12 years)	86	28.7		Duration of chronic pain		
Graduate/Bachelor's degree	68	22.7		Less than 6 months	74	24.7
Postgraduate/Master's degree or above	24	8.0		6 months-1 year	72	24.0
Occupation				1-3 years	71	23.7
Student	69	23.0		3-5 years	51	17.0
Employed	72	24.0		More than 5 years	32	10.7
Home-maker	73	24.3		Type of chronic pain		
Unemployed	44	14.7		Musculoskeletal/Back pain	57	19.0
Retired	42	14.0		Joint pain/Arthritis	93	31.0
Body mass index (BMI)				Headache/Migraine	106	35.3
Underweight (<18.5)	65	21.7		Neuropathic pain	44	14.7
Regular (18.5-24.9)	69	23.0				

Note. N = number of participants; f = frequency, % = percentage

### Longitudinal associations between pain severity, medication adherence risk, and self-reported kidney risk

Across all time points, pain severity, NSAID adherence risk, and self-reported kidney risk were positively and consistently correlated, indicating stable longitudinal relationships among the three measures (BPI r = 0.36-0.52; MARS r = 0.56; SRKR r = 0.53-0.62, p < 0.001; Table 2). An increased level of pain was linked to increased risk of medication non-adherence and increased perceived kidney risk, indicating that the two factors were still related during the 12-month follow-up.

**Table 2:** Spearman's correlation matrix for brief pain inventory, medication adherence risk scores, and self-reported kidney risk across four time points (N = 300)

Variable	1	2	3	4	5	6	7	8	9	10	11	12
Brief pain inventory (baseline)	—	0.42**	0.38**	0.36**	0.30**	0.33**	0.31**	0.29**	0.35**	0.37**	0.40**	0.39**
Brief pain inventory (3 months)	—	—	0.48**	0.45**	0.32**	0.36**	0.34**	0.33**	0.38**	0.41**	0.44**	0.43**
Brief pain inventory (6 months)	—	—	—	0.52**	0.30**	0.35**	0.33**	0.32**	0.39**	0.43**	0.47**	0.45**
Brief pain inventory (12 months)	—	—	—	—	0.28**	0.33**	0.31**	0.30**	0.36**	0.40**	0.43**	0.42**
Medication adherence risk scores (baseline)	—	—	—	—	—	0.55**	0.58**	0.60**	0.42**	0.45**	0.48**	0.47**
Medication adherence risk scores (3 months)	—	—	—	—	—	—	0.63**	0.61**	0.43**	0.46**	0.49**	0.48**
Medication adherence risk scores (6 months)	—	—	—	—	—	—	—	0.67**	0.46**	0.49**	0.52**	0.51**
Medication adherence risk scores (12 months)	—	—	—	—	—	—	—	—	0.48**	0.50**	0.54**	0.53**
Self-reported kidney risk (baseline)	—	—	—	—	—	—	—	—	—	0.53**	0.55**	0.54**
Self-reported kidney risk (3 months)	—	—	—	—	—	—	—	—	—	—	0.58**	0.57**
Self-reported kidney risk (6 months)	—	—	—	—	—	—	—	—	—	—	—	0.62**
Self-reported kidney risk (12 months)	—	—	—	—	—	—	—	—	—	—	—	—

Note. ***= *p* < 0.001 considered significant; Positive correlations indicate direct relationships, and negative correlations indicate inverse relationships; N = 300.

### Gender-based differences in pain severity, medication adherence risk, and self-reported kidney risk

Women reported significantly higher pain severity, greater NSAID medication adherence risk, and higher self-reported kidney risk compared to men (BPI mean rank 163.4 vs. 130.2, U = 8575.6, p < 0.001; MARS mean rank 160.1 vs. 135.1, U = 9583.8, p = 0.014; SRKR mean rank 156.2 vs. 142.8, U = 10388.4, p = 0.040; Table 3). Overall, females exhibited higher levels across all three outcomes.

**Table 3:** Gender differences in pain severity, medication adherence, and self-reported kidney risk based on Mann-Whitney U tests (N = 300)

Variable	Gender	N	Mean rank	Sum of ranks	U	Z	p
Total brief pain inventory	Male	118	130.2	15363.6	-	-	-
	Female	182	163.4	29738.8	8575.600	-3.65	< 0.001***
Total medication adherence risk score	Male	118	135.1	15941.8	-	-	-
	Female	182	160.1	29160.2	9583.800	-2.45	0.014*
Total self-reported kidney risk	Male	118	142.8	16850.4	-	-	-
	Female	182	156.2	28452.6	10388.400	-2.05	0.040*

Note. N = 300 (Males = 118, 39.3%; Females = 182, 60.7%); Mann-Whitney U test was used for all comparisons; p values marked with *, *** indicate statistical significance at p < 0.05, < 0.001

### Age-group differences in pain severity, medication adherence risk, and self-reported kidney risk

Older participants reported significantly higher pain severity, greater NSAID medication adherence risk, and higher self-reported kidney risk compared to younger participants (Kruskal-Wallis: pain χ^2^(4) = 28.56, p < 0.001; adherence χ^2^(4) = 24.32, p < 0.001; kidney risk χ^2^(4) = 33.14, p < 0.001; Table 4). The youngest group (18-29 years) had the lowest scores across all outcomes, while the oldest groups (50-59 years and ≥ 60 years) exhibited the highest levels. Overall, age was positively associated with all three measures.

**Table 4:** Age group differences in pain severity, medication adherence, and self-reported kidney risk based on Kruskal-Wallis tests (N = 300)

Variable	Age group	N	Mean rank	x^2^ (df=4)	p
Total brief pain inventory	18-29 years	20	95.4	-	-
	30-39 years	22	135.55	-	-
	40-49 years	47	168.8	-	-
	50-59 years	95	172.45	-	-
	60+ years	116	170.1	28.56	< 0.001***
Total medication adherence risk score	18-29 years	20	88.3	-	-
	30-39 years	22	142.6	-	-
	40-49 years	47	160.9	-	-
	50-59 years	95	165.5	-	-
	60+ years	116	155.1	24.32	< 0.001***
Total self-reported kidney risk	18-29 years	20	75.2	-	-
	30-39 years	22	140.4	-	-
	40-49 years	47	152.3	-	-
	50-59 years	95	168.8	-	-
	60+ years	116	172.25	33.14	< 0.001***

Note. N = number of participants in each age category; % = percentage of the total sample; Percentages are based on total N = 300; Values are mean ranks from Kruskal-Wallis H tests; Overall test statistics are reported in the bottom row for each dependent variable: Total BPI (χ^2^(4) = 28.56, < 0.001***), Total MARS (χ^2^(4) = 24.32, < 0.001***), and Total SRKR (χ^2^(4) = 33.14, < 0.001***); Significance levels: p < 0.001***

### Temporal changes and within-subject effects of pain severity, medication adherence risk, and self-reported kidney risk over 12 months

Over the 12-month follow-up, pain severity (BPI), NSAID adherence risk (MARS), and self-reported kidney risk (SRKR) increased slightly but consistently (Baseline M = 14.00 ± 1.65; 12 months M = 14.25 ± 1.63; Table 5). Repeated-measures ANOVA showed significant within-subject effects for time and interactions with all measures (F = 3.12-42.18, p < 0.001; partial η^2^ = 0.014-0.051), indicating that these outcomes remained interconnected and changed steadily over the four time points.

**Table 5:** Descriptive statistics and Within-Subjects effects for brief pain inventory (BPI), medication adherence risk score (MARS), and self-reported Kidney Risk across time points (Baseline, 3 months, 6 months, 12 months; N = 300)

SRKR time	BPI time	MARS time	M	SD	N	-
Self-reported kidney risk (baseline)	Brief Pain Inventory (Baseline)	Medication Adherence Risk Scores (Baseline)	14	1.65	1200	-
	Brief Pain Inventory (3 months)	Medication Adherence Risk Scores (3 months)	14.02	1.64	1200	-
	Brief Pain Inventory (6 months)	Medication Adherence Risk Scores (6 months)	14.05	1.63	1200	-
	Brief Pain Inventory (12 months)	Medication Adherence Risk Scores (12 months)	14.1	1.62	1200	-
Self-reported kidney risk (3 months)	Brief Pain Inventory (Baseline)	Medication Adherence Risk Scores (Baseline)	14.05	1.63	1200	-
	Brief Pain Inventory (3 months)	Medication Adherence Risk Scores (3 months)	14.08	1.62	1200	-
	Brief Pain Inventory (6 months)	Medication Adherence Risk Scores (6 months)	14.12	1.61	1200	-
	Brief Pain Inventory (12 months)	Medication Adherence Risk Scores (12 months)	14.18	1.6	1200	-
Self-reported kidney risk (6 months)	Brief Pain Inventory (Baseline)	Medication Adherence Risk Scores (Baseline)	14.1	1.64	1200	-
	Brief Pain Inventory (3 months)	Medication Adherence Risk Scores (3 months)	14.12	1.63	1200	-
	Brief Pain Inventory (6 months)	Medication Adherence Risk Scores (6 months)	14.16	1.62	1200	-
	Brief Pain Inventory (12 months)	Medication Adherence Risk Scores (12 months)	14.2	1.61	1200	-
Self-reported kidney risk (12 months)	Brief Pain Inventory (Baseline)	Medication Adherence Risk Scores (Baseline)	14.15	1.66	1200	-
	Brief Pain Inventory (3 months)	Medication Adherence Risk Scores (3 months)	14.18	1.65	1200	-
	Brief Pain Inventory (6 months)	Medication Adherence Risk Scores (6 months)	14.22	1.64	1200	-
	Brief Pain Inventory (12 months)	Medication Adherence Risk Scores (12 months)	14.25	1.63	1200	-
Source	Type III SS	df	Mean square	F	p	Partial η^2^
Time	225.32	3	75.11	42.18	<0.001***	0.051
Time × brief pain inventory	18.42	9	2.05	8.12	<0.001***	0.019
Time × medication adherence risk scores	20.52	9	2.28	9.01	<0.001***	0.022
Time × brief pain inventory × medication adherence risk scores	14.42	27	0.534	3.12	<0.001***	0.014
Error (time)	253504.48	57552	4.40	-	-	-

Note. SD = standard deviation; N = number of participants; SS = sum of squares; BPI = Brief Pain Inventory; MARS = Medication Adherence Risk Scores; SRKR = Self-Reported Kidney Risk; ***p < 0.001; Partial η^2^ represents effect size.

## Discussion

This 1-year cohort study analysed the relationship between the level of chronic pain and NSAID- associated medication adherence and self-reports of kidney risk indicators in adults with persistent pain. Higher chronic pain was consistently linked with greater medication non-adherence at all time points. This is in line with the previous evidence that pain intensity is a significant determinant of non-adherence and that it is closely associated with both underuse and overuse of analgesics^12)^. Self- reported kidney risk was also related to higher medication non-adherence risk, supporting prior findings that chronic NSAID use increases perceived kidney risk^13)^. We have found that pain severity is moderately correlated with perceived kidney risk, which is supported by the fact that pain is persistent among patients with CKD, especially those on dialysis or non-dialysis CKD^14)^. This implies that more pain might make individuals more aware of kidney vulnerability. Still, the behaviours influenced by medication appear to have a more substantial effect on perceived kidney risk.

In the present study, female participants reported a pain severity level that was considerably higher than that of males, consistent with studies showing that women in chronic pain populations often experience more intense pain^15)^. Likewise, women had a greater risk for medication non-adherence, which confirms the results that women generally show lower adherence to the prescribed treatments because of sociodemographic and psychological reasons^16)^. The self-perceived kidney risk was a little bit higher in women, which is in line with the earlier studies that reported higher CKD prevalence, symptom burden, and women's inclination towards conservative treatment and thus possibly increasing their awareness and perceived susceptibility^17)^.

Age was a significant factor in our research, with older people reporting higher pain scores on the Brief Pain Inventory than younger people. This finding is consistent with longitudinal studies that uncover the higher prevalence and continuity of chronic pain in older people, hence underscoring the need for pain management in this population^18)^. Non-adherence was most pronounced amongst participants aged 50-59 years, which is consistent with the evidence where low self-efficacy and poor medication literacy are contributors to non-adherence in the elderly^19)^. Self-reported kidney risks also rose with age, consistent with previous research showing that older adults considered themselves more vulnerable to chronic kidney disease and its risk factors owing to greater knowledge^20)^. It should be noted that these results represent associations, not causal relationships. Severity of pain, adherence behaviour, and perceived kidney risk may also be affected by other factors, namely, comorbidities, polypharmacy, or medication burden.

Over the 12 months, the self-reported kidney risk in our study gradually increased, suggesting that participants' perception of kidney risk also increased. Likewise, in a similar survey of pre-dialysis CKD patients, illness perceptions, including perceived kidney vulnerability, were significant and changed over time, indicating the dynamic nature of patients' risk awareness^21)^. Over time, both groups of participants—the ones with higher medication adherence risk and the ones with greater pain scores—reported greater self-perceived kidney risk, consistent with studies showing that NSAID use is associated with higher perceived kidney risk; besides, the higher pain load may make more people conscious of the dangers regarding kidney health^13, 14)^.

### Limitations and future directions

This investigation is not without its drawbacks. First, the reliance on self-reported data, including the non-validated self-reported kidney risk checklist and the adapted MARS, limits the precision with which actual kidney injury and medication adherence could be determined. No clinical renal biomarkers were measured, and NSAID type, dose, and treatment duration were not differentiated. Consequently, the findings reflect perceived kidney risk and adherence behaviours rather than confirmed clinical outcomes. Also, the predominantly older sample might limit generalisation. Therefore, future research should incorporate laboratory markers, validate community-level kidney risk tools, analyse specific NSAID patterns, and test educational or behavioural interventions to encourage safer medication use. Additionally, more studies should be done to reveal the psychosocial factors that affect pain and adherence, to pinpoint high-risk groups, and thus, to make the process of early detection and prevention of renal complications more efficient.

## Conclusion

This study followed a group of participants over one year. It showed that chronic pain intensities, behaviours related to the use of NSAID medications, and self-reported kidney risk are all interconnected and remain constant over time. Chronic pain patients had consistently higher levels of poor compliance and renal risk perception, which showed an association with higher perceived kidney risk in patients who are undergoing long-term pain management. Female patients and older adults showed exceptionally high scores across all outcomes, indicating groups that may require more clinical attention.

Despite only minor changes in outcomes over time, the consistent upward trend indicates the potential for increasing self-reported kidney risk over time when chronic pain and long-term NSAID use coexist. These results reiterate the necessity of constant monitoring, educating patients, and applying early preventive tactics to safeguard the kidneys in the management of chronic pain.

## Data availability

The datasets generated and analysed during the current study are available from the corresponding author on reasonable request.

## Author contributions

AR and AH conceived and designed the study. GAA, NAAM, and SS contributed to data acquisition and verification. AS and MNK conducted the literature review and provided methodological input. SMMA and OAB critically reviewed the manuscript for clinical relevance and intellectual content. ZAHM and ZAHM assisted with manuscript drafting, referencing, and formatting. SG served as the corresponding author, coordinated the project, and finalized the manuscript. All authors read and approved the final version of the manuscript.

## Conflicts of interest statement

The authors declare that there are no conflicts of interest.
